# Exploratory Search for Characteristic Symptoms to Distinguish Meibomian Gland Dysfunction from Dry Eye in a Population-Based Study in Japan

**DOI:** 10.3390/jcm11061715

**Published:** 2022-03-19

**Authors:** Reiko Arita, Takanori Mizoguchi, Motoko Kawashima, Shima Fukuoka, Shizuka Koh, Rika Shirakawa, Takashi Suzuki, Naoyuki Morishige

**Affiliations:** 1 Lid and Meibomian Gland Working Group, 626-11 Minami-Nakano, Minumaku, Saitama 337-0042, Japan; motoko326@gmail.com (M.K.); fshima3271@gmail.com (S.F.); skoh@ophthal.med.osaka-u.ac.jp (S.K.); rikadream@hotmail.com (R.S.); takashisuzuki58@gmail.com (T.S.); morishig@corneajp.com (N.M.); 2 Department of Ophthalmology, Itoh Clinic, 626-11 Minami-Nakano, Minumaku, Saitama 337-0042, Japan; 3 Department of Ophthalmology, Mizoguchi Eye Clinic, 6-13 Tawaramachi, Sasebo 857-0016, Japan; 4 Department of Ophthalmology, Kuki Kawashima Eye Clinic, Keio University, 35 Shinanomachi, Shinjukuku, Tokyo 160-8582, Japan; 5 Department of Ophthalmology, Omiya Hamada Eye Clinic, 1-169-1 Sakuragicho, Omiyaku, Saitama 330-0854, Japan; 6 Department of Innovative Visual Science, Graduate School of Medicine, Osaka University, 2-15 Yamadaoka, Suita 565-0871, Japan; 7 Department of Ophthalmology, The University of Tokyo, 7-3-1 Hongo, Bunkyoku, Tokyo 113-8655, Japan; 8 Department of Ophthalmology, Toho University Omori Medical Center, 6-11-1 Omorinishi, Otaku, Tokyo 143-8541, Japan; 9 Division of Cornea and Ocular Surface, Ohshima Eye Hospital, 11-8 Kamigofukumachi, Hakataku, Fukuoka 812-0036, Japan

**Keywords:** meibomian gland dysfunction, dry eye, meibomian gland, ocular symptom, population-based study, tearing sensation, meibography, meiboscore

## Abstract

Symptom overlap between meibomian gland dysfunction (MGD) and dry eye (DE) makes it difficult to distinguish between these two conditions on the basis of symptoms alone. We searched for characteristic symptoms that might help to distinguish MGD from DE on the basis of a population-based study. Subjects comprised 311 residents of Takushima island (18 to 96 years), including 117 individuals with MGD and 114 with DE. Responses to a symptom-related questionnaire (19 items) were subjected to factor analysis, and univariate regression analysis was performed to identify ocular surface parameters associated with characteristic symptoms of MGD. Factor analysis revealed aggregation of symptoms according to three factors: Factor 1 related to Symptom Score, Factor 2 to DE, and Factor 3 to MGD. Symptoms associated with DE included 11 items, whereas the only item related to MGD was tearing sensation. Pearson’s correlation analysis revealed that tearing sensation was associated with tear meniscus height (TMH), noninvasive tear-film breakup time, fluorescein staining score, meiboscore, meibum grade, and Schirmer value. Subjects with MGD experienced significantly more tearing and had a larger TMH than did those without MGD (*p* = 0.0334). Tearing sensation may thus be a characteristic symptom of MGD. Physicians should suspect MGD who complain of tearing sensation.

## 1. Introduction

Hippocrates of Kos based his practice of medicine on interviewing patients with regard to their symptoms [1]. Despite the availability today of high-tech devices for disease diagnosis, asking a patient about his or her symptoms provides both an opportunity for a physician to connect with the patient as well as important clues for diagnosis. Meibomian gland dysfunction (MGD) is one of the most common diseases encountered in ophthalmology clinics but has often been overlooked as a result of the similarity of its symptoms to those of dry eye (DE).

In 2011, the report of an international workshop organized by the Tear Film and Ocular Surface Society (TFOS) defined MGD as “a chronic abnormality of meibomian glands characterized by terminal duct obstruction or qualitative or quantitative changes in the glandular secretion, which can result in alteration of the tear film, inflammation, ocular surface disease, and symptoms of eye irritation” [2]. MGD as diagnosed on the basis of plugging of gland orifices and lid margin telangiectasia was found to be present in 61.7% of patients with ocular symptoms [3]. Other studies similarly found that 63.6% [4] or 64.6% [5] of such symptomatic individuals had signs of MGD. In addition, we previously proposed that the diagnostic criteria for obstructive MGD include the presence of at least one DE-related symptom, at least one lid margin abnormality, and a meiboscore as determined by noncontact meibography of >3 [6]. Such previous studies have indicated that ocular symptoms are a key factor for the diagnosis of MGD.

Questionnaires such as the Ocular Surface Disease Index (OSDI), Standard Patient Evaluation of Eye Dryness (SPEED), and McMonnies Dry Eye Questionnaire allow the assessment of a range of symptoms associated with ocular discomfort. However, given that many such symptoms are common to a range of disorders including DE and MGD [7], these questionnaires are unlikely to be able to differentiate between such etiologically distinct entities [8]. These questionnaires have thus not been optimized or tested for the ability to differentiate between MGD and other causes of ocular discomfort. The MGD workshop report of 2011 concluded that further studies are necessary in particular to assess the ability of specific symptom-based questionnaires to diagnose defined MGD patients as well as to discriminate MGD from DE and other related conditions on the basis of a pathognomonic symptom [8]. The SPEED score [9] was shown in one study to be correlated with clinical measures of meibomian gland function [10], but the subjects enrolled in this study were DE patients [10]. Furthermore, although a specific symptom-based questionnaire for MGD was developed, it was not able to differentiate MGD from DE [11].

On the basis of the results of a population-based study in Japan (Hirado–Takushima study), we recently showed that, whereas the symptoms of MGD and those of DE were similar, the risk factors, etiology, and pathogenesis of these two conditions were different [12]. In this previous study, we found that male sex, age, and the use of lipid-lowering agents were significantly associated with MGD, whereas female sex, contact lens wear, and the presence of conjunctivochalasis or lid margin abnormalities were significantly associated with DE. Although MGD has attracted attention as a leading cause of DE [2,13], it is also considered to be a cause of posterior blepharitis [2,14]. Given that ocular symptoms are an important cue for diagnosis in ophthalmology clinics and that MGD and DE are managed differently, exploration of symptoms able to distinguish between these two conditions should be pursued. As the development of a characteristic symptom questionnaire based on the pathology of MGD is likely necessary for discrimination between MGD and DE, we have now explored potential pathognomonic symptoms capable of differentiating MGD from DE on the basis of the results of our population-based study.

## 2. Materials and Methods

### 2.1. Subjects

This population-based cross-sectional study complied with the tenets of the Declaration of Helsinki and was approved by the Institutional Review Board of Itoh Clinic and registered in the University Hospital Medical Information Network database (UMIN 000028310). Written informed consent was obtained from all subjects before inclusion in the study [12].

### 2.2. Questionnaires

Study participants completed questionnaires covering general information, systemic conditions, and ocular symptoms, the latter of which were addressed by the DEQS questionnaire [15] and an additional four questions—concerning tearing sensation, feeling of discharge, itchy sensation, and oppressive sensation—that have been included in previous clinical studies by LIME [6,16]. These additional four items were selected from 14 questionnaire items—tired eyes, feeling of discharge, grittiness, dryness, uncomfortable sensation, sticky sensation, ocular pain, tearing sensation, itchy sensation, redness, oppressive sensation, glare, excessive blinking, and history of chalazion or hordeolum—previously adopted by LIME and do not overlap with the DEQS questionnaire [16]. The frequency of the DEQS questionnaire was graded from 0 to 4, with 0 corresponding to never, 1 to occasionally, 2 to sometimes, 3 to often, and 4 to always (Table 1). The frequency of the four items was graded from 0 to 3, with 0 corresponding to never, 1 to occasionally, 2 to sometimes, and 3 to always (Table 1).

### 2.3. Examinations

Assessments of the anterior segment of both eyes were performed according to standardized protocols by a team of seven ophthalmologists with expertise in DE and MGD (LIME members). TMH was measured with the Keratograph 5 M instrument (Oculus, Wetzlar, Germany); the thickness of the lipid layer of the tear film was determined with the LipiView interferometer (Johnson and Johnson, Stamford, CT, USA); lipid layer grade and noninvasive breakup time of the tear film were assessed with the DR-1α interferometer (Kowa, Aichi, Japan) [17]; lid margin abnormalities [18]—including plugging (scale of 0–3), vascularity (0–3), displacement of the mucocutaneous junction (0–3), and irregularity (0–3)—as well as fluorescein-based breakup time of the tear film, corneal–conjunctival fluorescein staining score (0–9) [19], the absence or presence of conjunctivochalasis, pterygium, conjunctival papillae, and *Demodex* mites, and the grading of meibum expressed with digital pressure (0–3) [20] were evaluated by slitlamp microscopy; the meiboscore (0–3 for each eyelid), which reflects the morphology of meibomian glands, was determined with a noncontact meibography system (Topcon, Tokyo, Japan) [21]; and the volume of tear fluid was measured by Schirmer’s test without the administration of anesthetic.

### 2.4. Definition of MGD

MGD was defined [22] as (1) any chronic ocular symptom [15]; (2) more than one lid margin abnormality among vascularity, displacement of the mucocutaneous junction, and irregularity; and (3) obstruction of meibomian glands as revealed by the detection of plugging and reduced meibum expression in response to moderate digital pressure [20]. The ophthalmologists performing examinations were masked to the results of tests performed by others.

### 2.5. Definition of DE

DE was defined according to the Japanese criteria of (1) the presence of any DE symptom [15] and (2) a fluorescein-based breakup time of the tear film of ≤5 s [23]. The definition of DE is thus independent of meibum quality or tear secretion, encompassing individuals with normal or abnormal meibomian gland function.

### 2.6. Statistical Analysis

The relation of ocular symptoms (as determined with the DEQS questionnaire and an additional questionnaire addressing another four items) of patients with MGD to those of patients with DE was examined by factor analysis in an attempt to identify characteristic symptoms related to each condition. ROC curve analysis was performed to test the discrimination ability of characteristic symptoms for MGD. The relation of ocular surface parameters to characteristic symptoms for MGD was evaluated by Pearson’s correlation coefficient analysis. The relation of TMH to tearing sensation was investigated with a linear mixed-effects model for subjects with or without MGD. For the factor analysis, variables with a *p*-value of <0.1 on univariate logistic analysis were selected as candidates for factors that might influence symptoms characteristic of MGD. Otherwise, a *p*-value of <0.05 was considered statistically significant. All statistical analysis was performed by an independent statistician with the use of SAS software version 9.4 (SAS Institute, Cary, NC, USA).

## 3. Results

### 3.1. Subjects

The demographics of the study subjects are shown in Table 2. A total of 384 individuals out of 628 residents of Takushima island agreed to participate in the population-based study. Individuals who were younger than 18 years of age (*n* = 45), who were unable to use a chin rest for ocular examinations, who terminated their participation, or who were unable to understand the procedure (*n* = 28) were to be excluded from the present study. Individuals unable to give informed consent as well as those who had sustained ocular trauma or undergone ophthalmic surgery in the previous 3 months were also not to be included in the study. A total of 311 individuals (110 males, 201 females), with a mean ± s.d. age of 60.8 ± 16.4 years (range, 18 to 96 years), were finally included in the study. None of the subjects with DE had Sjögren’s syndrome.

### 3.2. Relation between MGD Symptoms and DE Symptoms

**Figure 1:**
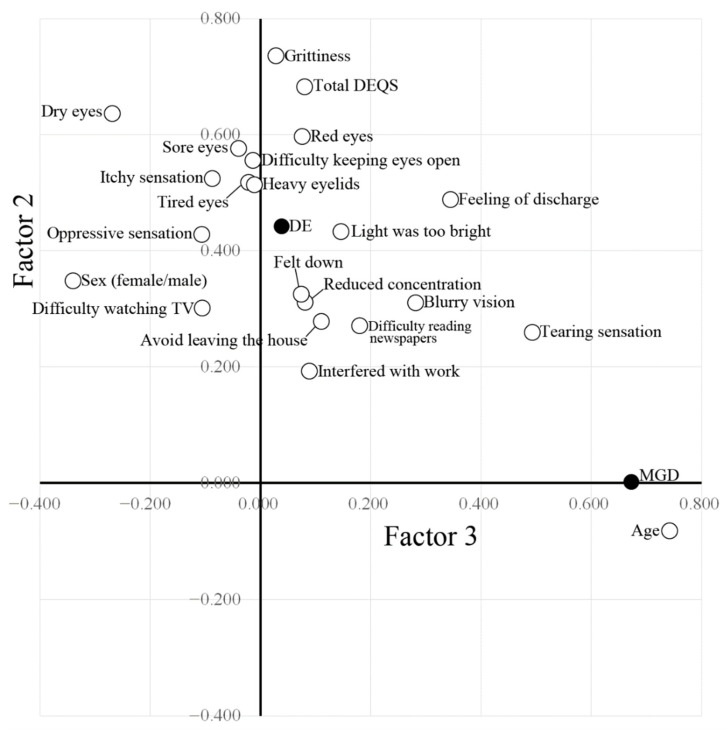
Factor analysis. Factor 2, which is related to dry eye (DE), is plotted against Factor 3, which is related to meibomian gland dysfunction (MGD). Black circles indicate diagnosis of DE or MGD in both eyes. Whites circles indicate age, sex and ocular symptoms.

Factor analysis for MGD patients (*n* = 117) and DE patients (*n* = 114) revealed aggregation of symptom items according to three factors: Factor 1 related to Dry Eye–Related Quality-of-Life Score (DEQS), Factor 2 to DE, and Factor 3 to MGD (Table 3). The parameters contributing most to MGD were age followed by tearing sensation. On the other hand, the parameters contributing most to DE were grittiness, total DEQS, ocular dryness, ocular redness, ocular pain, difficulty keeping eyes open, itchy sensation, tired eyes, heavy eyelids, feeling of discharge, glare, and oppressive feeling (Table 3, Figure 1). Although DEQS (Factor 1) and DE (Factor 2) were well correlated (*r* = 0.469), DEQS (Factor 1) and MGD (Factor 3) were not (*r* = 0.071) (Table 4). Moreover, DE (Factor 2) and MGD (Factor 3) were also not related (*r* = −0.025).

### 3.3. Discrimination of MGD on the Basis of Tearing Sensation

Receiver operating characteristic (ROC) curve analysis revealed that the area under the curve (AUC) for the diagnosis of MGD on the basis of tearing sensation was 0.689. Sensitivity was 65.8% (77/117) and specificity was 63.7% (123/193).

### 3.4. Ocular Surface Parameters Related to Tearing Sensation

**Figure 2:**
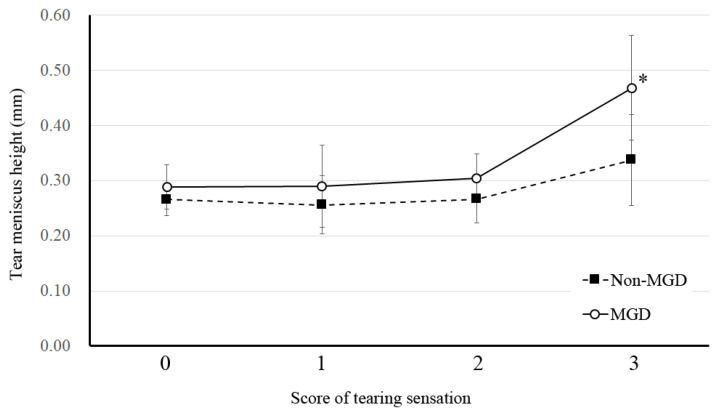
Relation between tear meniscus height (TMH) and frequency of tearing sensation in subjects with or without MGD. Data for TMH are means ± s.d. *p*-values were determined with a linear mixed-effects model (* *p* < 0.05).

Pearson’s coefficient analysis revealed a significant positive association between tearing sensation and tear meniscus height (TMH), noninvasive breakup time of the tear film, meiboscore, meibum grade, and Schirmer test value (Table 5). Fluorescein staining score and lipid layer condition type 1 (Jupiter-like appearance) showed a significant negative association with tearing sensation (Table 5). A linear mixed-effects model showed that MGD patients experienced a higher frequency of tearing sensation and had a significantly larger TMH than did individuals without MGD, with the difference in mean values being 0.130 mm (95% confidence interval of 0.010–0.250, *p* = 0.0334) (Figure 2).

## 4. Discussion

In the present study, factor analysis suggested the possibility of tearing sensation as a pathognomonic symptom for differentiation of MGD from DE. Univariate regression analysis revealed a significant positive association of tearing sensation with parameters related to the volume of tear fluid or to dysfunction of meibomian glands. These results likely reflect increased production of tear fluid to compensate for MGD and thereby to maintain homeostasis of the tear film [16,17]. In ophthalmology clinical practice, poor tear pump function, obstructive lacrimal drainage disorders, neurogenic lacrimal hypersecretory disorders, or inflammatory conditions of the ocular surface are suspected in patients who complain of tearing sensation [24]. Our results now suggest that MGD should also be suspected in such patients. Representative symptoms such as tearing sensation thus warrant inclusion together with meiboscore, lid margin abnormalities, and meibum grade as the most appropriate clinical parameters for diagnosis of MGD.

We found that the frequency of ocular fatigue (65.0%), feeling of discharge (63.2%), itchy sensation (58.9%), and tearing sensation (57.2%) was high for MGD patients. On the other hand, the frequency of ocular fatigue (79.8%), blurry vision (66.7%), feeling of discharge (65.7%), and itchy sensation (63.2%) was high for DE patients. The frequency of ocular fatigue, feeling of discharge, and itchy sensation was thus high in both MGD patients and DE patients. DE-related symptom questionnaires such as the OSDI [25], McMonnies questionnaire [26], Schein’s questionnaire [27], and DEQS [15] were adopted in previous population- or clinic-based studies for estimation of the prevalence of MGD [28], given the lack of an available MGD-specific questionnaire. In the present study, we applied a combination of the DEQS questionnaire [15] and four items of a Lid and Meibomian Gland Working Group (LIME) questionnaire previously used for MGD studies [6,16]. As most of ocular symptoms and signs tend to overlap between MGD and DE, this study tried exploratorily to search for characteristic symptoms for MGD. Exploratory factor analysis is often used in the multidimensional situation where more than one latent variable is measured at the same time. We would like to use the most collected data from our epidemiological study. Therefore, we chose exploratory factor analysis rather than simple statistical comparisons or correlation analysis of two groups. Factor analysis showed that DEQS was not related to MGD, although DEQS was able to account well for DE symptoms. Moreover, one of the four LIME items, tearing sensation, was found to be significantly associated with MGD. Although factor analysis implicated tearing sensation as a characteristic symptom of MGD separate from DE, the AUC value was not sufficiently high for tearing sensation to be considered a specific symptom of MGD that completely distinguishes MGD from DE. We reported that the risk factors, etiology, and pathogenesis of MGD and DE were different, although the symptoms of two conditions were similar according to the population-based study (Hirado–Takushima Study) [12]. Although MGD and DE sometimes coexist, the treatments for these two conditions differ, and therefore it is important to suspect and correctly diagnose MGD in patients with this condition. Our identification of tearing sensation as a characteristic symptom that can contribute to the differentiation of MGD from DE should thus prove clinically helpful.

The SPEED score was previously shown to be correlated with clinical measures of meibomian gland function [10], although the subjects enrolled in this previous study were DE patients [10]. The 5-Item Dry Eye Questionnaire (DEQ-5) was validated for discrimination between non-DE patients and aqueous-deficient DE patients [29]. The SPEED questionnaire and DEQ-5 are the only validated questionnaires for DE that include tearing (watery) sensation and may therefore be the most appropriate such questionnaires to detect MGD and aqueous-deficient DE. A specific symptom questionnaire for MGD [11] was developed after Rasch analysis of Schein’s questionnaire [27], the SPEED score [9], and LIME questionnaire [6,16]. Although this new questionnaire does not include tearing sensation, it does include burning sensation. The SPEED questionnaire includes burning/watery sensation in one line.

Pearson’s coefficient analysis in the present study revealed a significant positive association of tearing sensation with TMH and Schirmer test value, both of which reflect aqueous volume of the tear film. Moreover, tearing sensation was associated with the meiboscore and meibum grade, both of which indicate dysfunction of meibomian glands, with the meiboscore reflecting the lost area of meibomian glands in both upper and lower eyelids as detected by noninvasive meibography [21]. These results thus suggest that tear fluid production was increased as a compensatory response to meibomian gland loss [16,17]. Tearing sensation also showed a significant negative association with lipid layer condition type 1 (Jupiter-like appearance) [17] as visualized with the DR-1α interferometer. This condition corresponds to a thin aqueous layer and thick lipid layer of the tear film, and its negative association with tearing sensation is thus also consistent with the operation of a compensatory mechanism to maintain homeostasis of the tear film [17]. Subjects with MGD who complained of a tearing sensation had a larger TMH compared with those who did not. Of note, these results from our population-based study [12] correspond well to the findings of previous clinical studies showing that TMH was correlated with clinical parameters in patients with MGD [30,31,32].

We have previously investigated the balance between the lipid layer and the aqueous layer of the tear film [16,17], with our results having suggested that deficiencies in these layers trigger reciprocal compensatory responses in order to maintain homeostasis of the tear film [16,17]. The latest definition of DE by the TFOS Dry Eye Workshop Report II states that “Dry eye is a multifactorial disease of the ocular surface characterized by a loss of homeostasis of the tear film, and accompanied by ocular symptoms, in which tear film instability and hyperosmolarity, ocular surface inflammation and damage, and neurosensory abnormalities play etiological roles” [33]. With regard to homeostasis of the tear film, our results identifying tearing sensation as a differential symptom of MGD relative to DE seem consistent with the physiology and pathology of the tear film.

A recent clinic-based study of 2346 patients with DE symptoms in Singapore found that MGD and a lower forniceal papillary reaction contributed significantly to symptom severity [34]. DE symptoms, reduced meibomian gland expression, and inferior papillae associated with allergic factors including a history of allergic tendencies were strongly correlated in this previous study [34]. Pearson’s coefficient analysis in the present study revealed that tearing sensation was not associated with a history of systemic allergy (*p* = 0.1653) but showed a trend toward an association with conjunctival papillae (*p* = 0.0887). The present study was not clinic based with a large number of patients, however, but was population based with a relatively small number of subjects. Further studies are necessary to investigate the relations among DE, MGD, and allergic conjunctivitis.

There are some limitations to our study. First, given its population-based design and reliance of the study on a medical examination, we were not able to perform a nasolacrimal duct flow test. It is therefore possible that individuals with nasolacrimal disorders were included. Second, although we identified tearing sensation as a characteristic symptom for MGD, the specificity and sensitivity of this symptom for diagnosis of MGD will require validation in clinical practice. Future studies are also needed to establish diagnostic criteria for MGD that incorporate a subjective symptom questionnaire including tearing sensation as a representative symptom as well as objective findings such as the meiboscore, lid margin abnormalities, and meibum grade. Third, the population of Takushima island spends less time indoors and in front of screens in comparison with more urban populations, and it includes a lower proportion of contact lens wearers. These differences may limit the ability to extrapolate our results to the general population [12].

In conclusion, we have suggested the possibility of tearing sensation as a characteristic symptom for MGD, particularly with regard to the differentiation of MGD from DE. The clinical data obtained by our population-based study also provide further evidence for a compensatory response of the tear film to deficiencies in the lipid layer due to MGD. Further studies with healthy individuals as well as DE and MGD (mild, moderate, and severe stages) patients are needed to validate the diagnostic efficacy for MGD of a symptom questionnaire including tearing sensation. We propose that MGD should be suspected as a possible diagnosis and addressed when patients complain of tearing sensation.

## 5. Conclusions

Tearing sensation may thus be a characteristic symptom of MGD. Physicians should suspect MGD who complain of tearing sensation.

## Figures and Tables

**Table 1:** Dry Eye–Related Quality-of-Life Score (DEQS) questionnaire and an additional four symptom items about which subjects were asked.

	Never	Occasionally	Sometimes	Often	Always
(1) Grittiness (sensation of something in your eye)	0	1	2	3	4
(2) Dry eyes	0	1	2	3	4
(3) Sore eyes	0	1	2	3	4
(4) Tired eyes	0	1	2	3	4
(5) Heavy eyelids	0	1	2	3	4
(6) Red eyes	0	1	2	3	4
(7) Difficulty keeping my eyes open (due to my symptoms)	0	1	2	3	4
(8) Vision became blurry when engaging in activities that required sustained visual attention (e.g., computer working, reading, knitting)	0	1	2	3	4
(9) Light was too bright	0	1	2	3	4
(10) Eye symptoms worsened when reading newspapers, magazines, or books	0	1	2	3	4
(11) Eye symptoms worsened when watching TV or when using a computer/mobile phone	0	1	2	3	4
(12) Eye symptoms reduced my ability to concentrate	0	1	2	3	4
(13) Eye symptoms interfered with work, housework, or studying	0	1	2	3	4
(14) Tended to avoid leaving the house because of eye symptoms	0	1	2	3	4
(15) Felt down due to eye symptoms	0	1	2	3	4
(1) Tearing sensation	0	1	2	3	-
(2) Feeling of discharge	0	1	2	3	-
(3) Itchy sensation	0	1	2	3	-
(4) Oppressive sensation	0	1	2	3	-

**Table 2:** Demographics of the study subjects according to diagnoses of meibomian gland dysfunction (MGD) and dry eye (DE).

	*n*	Percentage	Mean Age ± s.d. (Age Range), Years
MGD (+)	117	37.6	67.5 ± 13.4 (28–96)
MGD (−)	194	62.4	56.7 ± 16.7 (18–90)
DE (+)	114	36.7	61.3 ± 16.9 (25–96)
DE (−)	197	63.3	60.5 ± 16.1 (18–90)
MGD (+) DE (+)	42	13.5	
MGD (+) DE (−)	75	24.1	
MGD (−) DE (+)	60	19.3	
MGD (−) DE (−)	134	43.1	
Total	311	100	60.8 ± 16.4 (18–96)

**Table 3:** Relations among ocular symptoms, dry eye (DE), and meibomian gland dysfunction (MGD) according to Factors 1, 2, and 3, respectively.

Parameter	Factor 1	Factor 2	Factor 3
DE	0.112	**0.442**	0.039
MGD	0.037	0.001	**0.673**
Age	0.097	−0.083	**0.742**
Sex (female/male)	0.059	0.348	−0.340
Grittiness	**0.420**	**0.735**	0.028
Dry eyes	**0.444**	**0.636**	−0.269
Sore eyes	**0.566**	**0.576**	−0.039
Tired eyes	**0.698**	**0.518**	−0.021
Heavy eyelids	**0.627**	**0.514**	−0.011
Red eyes	0.353	**0.597**	0.077
Difficulty keeping eyes open	**0.529**	**0.555**	−0.013
Blurry vision	**0.650**	0.310	0.282
Light was too bright	**0.579**	**0.433**	0.146
Eye symptoms worsened when reading	**0.742**	0.271	0.181
Eye symptoms worsened when watching TV or using a computer/phone	**0.714**	0.301	−0.105
Eye symptoms reduced ability to concentrate	**0.806**	0.311	0.082
Eye symptoms interfered with work, housework, or studying	**0.770**	0.193	0.089
Tended to avoid leaving house due to eye symptoms	0.243	0.278	0.111
Felt down due to eye symptoms	**0.710**	0.325	0.074
Total DEQS	**0.898**	**0.682**	0.080
Teary sensation	0.257	0.259	**0.494**
Feeling of discharge	0.093	0.488	0.345
Itchy sensation	0.278	**0.524**	−0.087
Oppressive sensation	**0.404**	**0.428**	−0.106

Values in bold indicate >0.40. DEQS, Dry Eye–Related Quality-of-Life Score.

**Table 4:** Correlation between factors in factor analysis (*n* = 308).

	Factor 1	Factor 2	Factor 3
Factor 1	1.000	0.469	0.071
Factor 2	0.469	1.000	−0.025
Factor 3	0.071	−0.025	1.000

**Table 5:** Pearson’s coefficient analysis of the relation between ocular surface parameters and teary sensation.

	*n*	Correlation Coefficient	Estimated Regression Coefficient	Lower Limit of 95% CI	Upper Limit of 95% CI	*p*-Value
**TMH (mm)**	**310**	**0.136**	**0.9320**	**0.1684**	**1.6956**	**0.0169**
Lipid layer thickness (nm)	310	0.043	0.0870	−0.1403	0.3143	0.4518
**Lipid layer condition** **(type 1, Jupiter-like)**	**259**	**−0.126**	**−0.2544**	**−0.5004**	**−0.0083**	**0.0428**
Lipid layer condition (type 2, crystal-like)	198	−0.028	−0.0692	−0.4155	0.2772	0.6941
**NIBUT (s)**	**307**	**0.130**	**0.0277**	**0.0038**	**0.0517**	**0.0232**
Plugging	310	0.015	0.0310	−0.1971	0.2590	0.7895
Vascularity, irregularity, displacement of MCJ	310	0.066	0.1804	−0.1233	0.4841	0.2435
FTBUT (s)	310	0.068	0.0335	−0.0219	0.0889	0.2349
**Fluorescein score**	**310**	**−0.141**	**−0.3193**	**−0.5699**	**−0.0687**	**0.0127**
Conjunctivochalasis	310	0.070	0.1865	−0.1105	0.4834	0.2176
**Pterygium**	**309**	**0.118**	**0.3604**	**0.0198**	**0.7010**	**0.0381**
Papilla formation	309	−0.097	−0.2060	−0.4434	0.0314	0.0887
**Meibum grade**	**310**	**0.149**	**0.3303**	**0.0849**	**0.5757**	**0.0085**
**Meiboscore**	**310**	**0.150**	**0.0899**	**0.0234**	**0.1564**	**0.0083**
*Demodex*	310	0.051	0.1026	−0.1248	0.3300	0.3753
**Schirmer test value**	**310**	**0.125**	**0.0115**	**0.0013**	**0.0217**	**0.0273**

Parameters in bold have a *p*-value of <0.05. CI, confidence interval; TMH, tear meniscus height; NIBUT, noninvasive breakup time of the tear film; MCJ, mucocutaneous junction; FTBUT, fluorescein-based breakup time of the tear film.

## Data Availability

The datasets generated during and analyzed in the current study are available from the corresponding author on request.
